# Examining palliative and end of life care research in Ireland within a global context: a systematic mapping review of the evidence

**DOI:** 10.1186/s12904-018-0364-7

**Published:** 2018-09-27

**Authors:** Sonja McIlfatrick, Deborah H. L. Muldrew, Felicity Hasson, Sheila Payne

**Affiliations:** 1 School of Nursing, Ulster University, Shore Road, Newtownabbey, Co Antrim BT37 0QB UK; 2 All Ireland Institute of Hospice and Palliative Care, Dublin, Ireland; 3 International Observatory on End of Life Care, Lancaster University, Lancaster, UK

**Keywords:** Palliative care, Systematic review, Research, Ireland

## Abstract

**Background:**

Globally the state of palliative care research remains uncertain. Questions remain regarding impact, funding, and research priorities. Building upon previous research, this review examines palliative care research in Ireland and contributes to a wider international debate on the state of palliative care research.

**Methods:**

A systematic mapping review was undertaken. Eight bibliographic databases and thesis repositories were searched from May 2012 to April 2017. Palliative care related search terms were combined with “Ireland” or “Irish” to increase search sensitivity. Inclusion criteria were applied by two independent reviewers. Descriptive analysis was completed using IBM SPSS v23. Thematic analysis was undertaken using a data-driven approach to develop new themes.

**Results:**

In total, 808 studies were screened and 151 papers from 117 studies were included for review. The top two areas of research focus included: (1) specific groups, services, and settings (*n* = 70); and (2) identification, communication and education (*n* = 37). A diverse variety of research methods were used including mixed methods (25%), surveys (22%), interviews (20%), and reviews (17%). One randomised control trial was conducted. The predominance of research papers focused solely on health care professionals (*n* = 35%), and the community setting was the most frequent location for data collection (41%). The majority of data was collected across the two jurisdictions of the Republic of Ireland (ROI) and Northern Ireland (NI) (37%), and 23% of studies included data outside of Ireland and the UK. The most frequent sources of funding were: consortiums (*n* = 40); government (*n* = 24); and philanthropic bodies (*n* = 20). Forty percent (*n* = 60) of papers were either unfunded or did not acknowledge a funder.

**Conclusions:**

There is a continued increase in palliative care research in Ireland with increased collaborative working nationally and internationally. The quantity and impact of research has increased from the previous review, which can be attributed to significant investment in research funding and collaborative networks. However, research gaps continue to exist including out of hours’ care, physical and psychological symptom control, intervention studies, and the patient and family perspective. Areas for attention include the need to ensure knowledge exchange and demonstrate impact of the research on patient and family carer outcomes.

**Electronic supplementary material:**

The online version of this article (10.1186/s12904-018-0364-7) contains supplementary material, which is available to authorized users.

## Background

A global increase in older people approaching the end of life with complex, chronic conditions has resulted in the need for robust palliative care research that will both enhance services and care [1]. Palliative care research aims to inform the core components of palliative care, as outlined in the World Health Organization’s (WHO) definition [2, 3], as well as many practical and economic aspects of delivery. Palliative care research is important to help inform policy and practice, for example, by influencing health care professional education, training, and government policy [4].

Whilst there have been significant developments in recent years, seeking to map and outline the situation of palliative care globally [5], this has not extended to explicitly outline the state of palliative care research. For example, Walshe [6] reported that whilst the latest European Association for Palliative Care (EAPC) Atlas of Palliative Care in Europe [7] does provide some information about research capacity in each country, this is limited and does not include information such as the type, quality, and quantity of research undertaken in each region. This information is needed to map out international palliative care research activity, which has been argued as a key approach to engage policymakers and influence healthcare organisations [8], thus improving the delivery of care.

This lack of global information around palliative care research capacity has led to individual countries seeking to assess the research they are undertaking. Recent reviews have sought to examine the state of palliative care research within specific regions, for example, Ireland [9], Scotland [10] Sweden [11], China [12], and South Asia [13], indicating an increased momentum for countries to take cognisance of the state of palliative care research in order to inform policy and practice, and also to inform the wider debate within an international context. The reviews to date indicated that while the quantity of palliative care research is increasing, it is often based on small, needs-based studies (Table 1). Similar patterns were evident in a recent mapping review of palliative care research in the context of global development [5]. This research indicated that “evaluation” and “views of stakeholders” were focal points of research, predominantly through the use of observational research. Interventional studies of effectiveness and cost effectiveness were largely absent, highlighting challenges when undertaking interdisciplinary research and when seeking to align the international research agenda with practice and policy.

**Table 1:** Summary of main findings from national reviews

Country	Date range	# of papers	Common focus	Common study groups	Common methodology	Missing research	Other observations
Ireland (McIlfatrick and Murphy, 2013)	2002–2012	151	Specific groups Services and settings Symptom management	Children Intellectual disability Malignant disease	Quantitative/Qualitative Mixed methods Systematic reviews Small sample, needs based	Public health Policy research	Upward trend in publication numbers
Scotland (Finucane et al., 2018)	2006–2015	308	Services and settings Experiences and/or needs Physical symptoms	Patients only Mixed groups HCPs only	Descriptive studies	E-health Health economics Out-of-hours Public health	Nearly half of all papers described unfunded research or did not acknowledge a funder
Sweden (Henoch et al., 2016)	2007–2012	263	Symptom assessment and management Experiences of illness Care planning	–	Small sample, cross sectional Qualitative	Ethnic minorities Non-verbally communicative people Children	Non-cancer populations and utilisation of population-based register studies identified as new features
China (Wang and Chan, 2015)	1991–2014	107	Attitudes to death Service utilisation Physical symptoms	In/ out -patients Older adults Care staff Informal caregivers	–	–	Research undertaken by healthcare professionals
South Asia (Singh & Hardin 2015)	1980–2013	16	Service description Intervention outcomes	Home care Care centre Hospital Outpatients	Descriptive Service evaluation (no (quasi) experimental designs)	Cultural context of death and dying for patients and families	Lack of data beyond India Urgent need for research investment

There has been a significant investment in palliative care research networks over the last five years across the two jurisdictions of Ireland (ROI and NI) with the establishment of the All Ireland Institute of Hospice and Palliative Care (AIIHPC) (www.aiihpc.org/) and Palliative Care Research Network (PCRN) [14]. These initiatives sought to build research capacity, provide research structural support, and act as a leverage for more funding through collaborative working. It was anticipated that these initiatives would increase research activity, however, the impact on the palliative care research agenda is unclear. This research builds upon the previous systematic review in Ireland [9], synthesizing palliative care research across Ireland published in the five year period between May 2012 and April 2017. This will help identify gaps in the literature, review the state of the science, inform policy and best practice in Ireland, and map this information globally.

## Methods

### Design

A systematic mapping review was undertaken to find both quantitative, qualitative, and mixed method published literature. Cooper [15] reports that mapping studies are based on the concept that published articles not only represent findings, but, indirectly, represent activity related to the finding. They seek to identify, not results, but linkages by focusing on characteristics such as where the activity took place, where the funding came from, and in what journal or other medium it was presented. Mapping, if done correctly, is on the higher reliability end of the spectrum of reviews. Grant and Booth [16] identify mapping reviews as a suitable method to map out and categorise existing literature as a means of identifying tendencies and gaps in the literature to commission future research.

### Search strategy

Eight bibliographic databases and thesis repositories were searched from May 2012 to April 2017: CINAHL; Embase; Medline; PsychInfo; Cochrane Palliative Care Database; EThOS; ProQuest; and RIAN. Key palliative care researchers in Ireland were also contacted via email. A combination of palliative care related search terms including “palliative,” “terminal,” “hospice”, “end of life”, “dying”, “death”, “bereavement”, and “grieving” were searched in combination with “Ireland” or “Irish” to increase the sensitivity of the search (Table 2).

**Table 2:** Medline search strategy

#	Search term	Hits
1	Palliative care mp.	7900
2	Hospice Care/	1051
3	Terminal Care/	4092
4	Hospice*	3489
5	Terminal*	80,544
6	Palli*	25,586
7	End of life.mp.	7477
8	dying	6890
9	Death/	1475
10	Bereavement/	874
11	Bereav*	2143
12	Grie*	3000
13	1 OR 2 OR 3 OR 4 OR 5 OR 6 OR 7 OR 8 OR 9 OR 10 OR 11 OR 12	115,235
14	Irish	2604
15	Ireland	2833
16	Northern Ireland	548
17	14 OR 15 OR 16	4888
18	13 AND 17	120
19	Limit 18 to english language and year 2012-current	94

^*^indication of truncation

Inclusion and exclusion criteria (Table 3) were applied to the titles and abstracts of the retrieved articles by two independent reviewers (DM and FH) prior to full text review. A quality appraisal was not undertaken in keeping with a mapping review [16], as the aim of this review was to describe and map the key areas of focus for palliative care research in Ireland over the past five years.

**Table 3:** Inclusion and Exclusion criteria

Inclusion criteria	
• Research focused on palliative and end of life care in accordance with the WHO definition of palliative care • Patients who i) are in the last year of life, or ii) have a terminal illness or iii) would benefit from palliative care, as well families, carers and health care professionals • Research with at least one author based at an Irish institution • Primary research, secondary research and literature reviews • Theses written as part of a higher degree (MD, PhD, MSc, DNP) • Service evaluation underpinned by research methods • English language • Peer reviewed and non-peer reviewed published research • Quality improvement projects underpinned by research methods • Palliative radiotherapy or chemotherapy where the aim is symptom management as opposed to disease modification • Research published between May 2012 and April 2017	
Exclusion criteria	
• Research based on data collected in Ireland where none of the authors were Irish-based • Research on disease modifying or active treatment • Grey literature including: Commentary papers; Editorials; Conference abstracts/proceedings; Service evaluation with no clear methodology; Audit; Case reports; Opinion pieces/letters; Guidelines/Guidance; Research protocols; Government publications; Reports; Policy documents; Statistical publications; Newsletters; Fact sheets; Working papers; and Technical reports • Research published before May 2012	

### Data extraction and analysis

All citations were exported to Mendeley and duplicates were removed. Data were extracted by two independent researchers (DM and FH) using a data extraction form, which had been piloted for appropriateness. Descriptive, statistical analysis on year of publication, journal, international co-authors, region of data collection, setting, population, methodology, design, methods, and funders was completed using IBM SPSS v23. Qualitative analysis on the title, key words, aim, results, and research themes consisted of re-reading, isolating, comparing, categorising and relating the data using a data-driven approach to form themes. Each article was allocated three themes, adapted from the previous review in Ireland by the research team, based on the key words and focus of results. These themes were used to form a narrative summary highlighting the key areas of research activity (See Additional file 1).

## Results

**Fig. 1:**
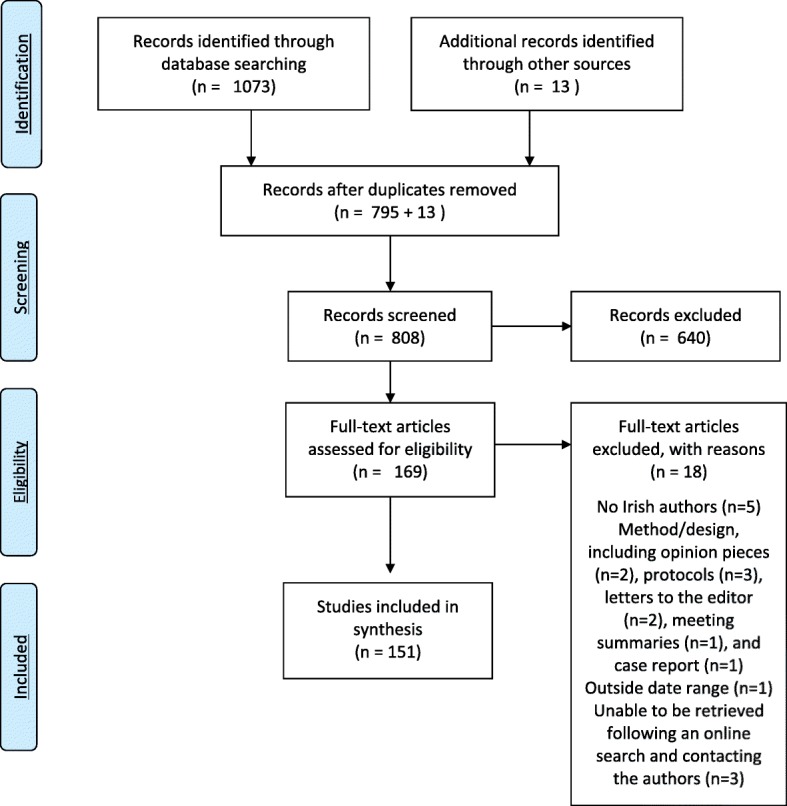
PRISMA Flow Diagram

A total of 1073 articles were retrieved from the database searches, and thirteen additional papers were identified via key contacts. Following the removal of duplicates, 808 titles and abstracts were screened against the inclusion criteria, resulting in 169 articles for full text review. Fifteen were excluded at full text review, and three were unable to be retrieved following an online search and contacting the authors, leaving 151 papers, reflecting 117 studies, to be included in the final analysis (Fig. 1). The main reasons for exclusion at full text review were: grey literature (*n* = 9); no Irish authors (*n* = 5); outside date range (*n* = 1); and unable to retrieved following an online search and contacting the authors (*n* = 3).

### Characteristics of selected papers

**Fig. 2:**
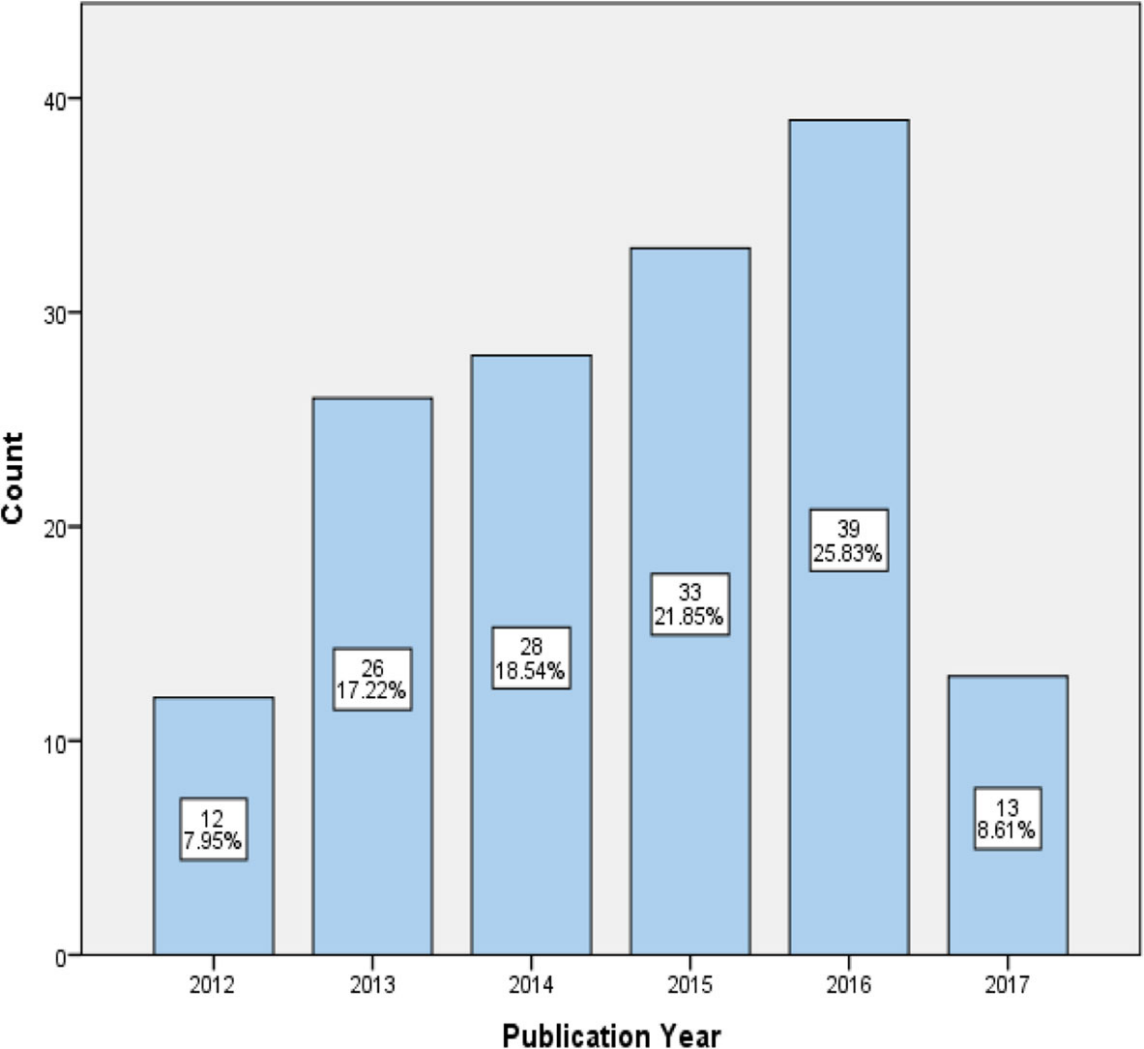
Number of publications per year (May 2012–April 2017)

In the five-year period (May 2012–April 2017), 151 papers were published from 117 studies which met the inclusion criteria, indicating a steady and sustained increased in research outputs (see Fig. 2).

**Fig. 3:**
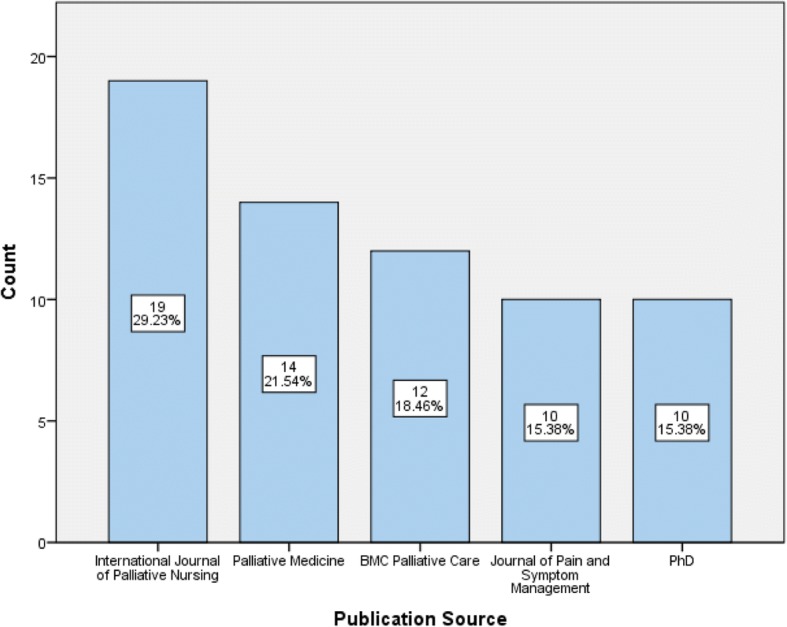
Top 5 most popular publication sources (2012–2017)

Papers were published in 53 national and international journals, the most popular being the International Journal of Palliative Nursing (30%), Palliative Medicine (22%), BMC Palliative Care (18%), and Journal of Pain and Symptom Management (15%). In addition, PhDs and MScs were available through online thesis repositories, and PhDs were the fifth most common publication type (15%) (see Fig. 3).

Source of funding was specified for 60% (*n* = 91). The majority of research (*n* = 40; 27%) was funded by a Consortium (e.g. Atlantic Philanthropies and Cicely Saunders Institute). Government funding (e.g. Health and Social Care Research and Development Office NI, Health Research Board Ireland) was the next most common source (*n* = 24; 16%), followed by philanthropic (e.g. Irish Hospice Foundation, Cancer Focus NI) funding (*n* = 20; 13%), and organisations (e.g. AIIHPC, EAPC) (*n* = 7; 5%). Nineteen papers (13%) were unfunded and 41 (27%) did not acknowledge a funder.

The majority of research used a quantitative (25%), or qualitative (23%) methodology. Common methods of data collection were mixed methods (25%), questionnaires, surveys, and standardised tools (22%), interviews or focus groups (20%), and reviews (17%). Other methods of data collection included retrospective case note reviews (6%), secondary data or database analysis (5%), expert panel (2%), participant observation (1%), randomised control trial (1%), and other (2%). Twenty-six papers did not undertake primary data collection, for example, systematic reviews. Of the remaining 125 papers, 35% recruited solely healthcare professionals (HCPs) to participate. Patients and carers were the second most commonly recruited population (30%), followed by a mixture of participants (18%), which could include HCPs, patients, carers, and other groups. Researchers (4%), students (4%), and the general public (2%) were rarely included.

The majority of data was collected across the two jurisdictions of ROI and NI (37%), whilst approximately a third of the studies were conducted within single regions of Ireland and NI. Developing international links were also evident, with 23% of studies including data collection outside of Ireland and the UK. Of the papers which collected primary data (*n* = 119), community settings were the most popular setting (41%), which included home care (*n* = 14), care homes (*n* = 10), hospice (*n* = 11), specialist palliative care units (*n* = 6), and General Practitioner (GP) practices (*n* = 4). This was followed by studies which used a mixture of settings for data collection (31%), which could have included community, hospital, or other settings. Almost a fifth of studies collected data in the hospital setting. Forty-one studies did not report the setting (See Table 4).

**Table 4:** Descriptive statistics

	Number	Percent
Design		
Quantitative	38	25.17
Qualitative	35	23.18
Mixed	30	19.87
Review	25	16.56
Secondary data analysis	14	9.27
Other	7	4.64
Service evaluation	2	1.32
Method		
Mixed/multi method	38	25.17
Questionnaire, surveys, and standardised assessment tools	33	21.85
Interviews or focus groups	30	19.87
Review	26	17.22
Case note review or documentary analysis	9	5.96
Secondary data analysis	4	2.65
Database analysis	3	1.99
Expert panel	3	1.99
Participant observation	1	0.66
Randomised control trial	1	0.66
Participants		
HCPs	44	35.20
Patients and carers	37	29.60
Mixed	22	17.60
Other	9	7.20
Researchers	5	4.0
Students	5	4.0
General public	3	2.40
Region		
Ireland/NI (National)	44	36.97
Ireland/NI (One region)	41	34.45
International excluding Ireland/NI	14	11.76
International including Ireland/NI	14	11.76
UK excluding Ireland/NI	4	3.36
UK including Ireland/NI	2	1.68
Setting		
Community (home care, care homes, hospice, specialised Palliative Care Units (PCUs))	45	40.91
Mixed	34	30.91
Hospital	20	18.18
Other	11	10.0

Primary research topics, identified through key terms, were non-cancer (*n* = 26), education, training and knowledge (*n* = 20), symptoms (*n* = 12), cancer (*n* = 12), and identification of needs (*n* = 10). Secondary research topics included experiences, perceptions, and needs (*n* = 30), methodology, assessment, and evaluation (*n* = 14), other (*n* = 5), last days of life (*n* = 8), and services and settings (*n* = 10).

### Thematic synthesis

Six core themes were identified after examination of the included articles, which were then mapped against the themes from the previous review of Irish palliative care research (Table 5).

**Table 5:** Comparison of core themes between reviews of research in Ireland

2002–2012 Themes	2012–2017 Themes
Specific groups/populations	Specific groups, services, and settings
Services and settings	
Management of symptoms	Symptom management
Bereavement	End of life care and bereavement
Death and dying	
Communication and education	Identification, communication and education
Complementary and alternative medicine/intervention	Experiences, perceptions, and needs
Spirituality	Methodology and Evaluation

The top two research themes were: Research focusing on specific groups, services, and settings (46%; *n* = 70); and identification, communication and education (25%; *n* = 37). Cancer and dementia were the most common diagnoses of interest, followed by Parkinson’s disease, and people with a disability (intellectual, learning, or neurodevelopmental). Services and settings focused mainly on hospice care (*n* = 5), however transition across multiple settings (*n* = 3), and home care (*n* = 1) were also included. Advance care planning (*n* = 3) and the Liverpool Care Pathway (*n* = 2) were the two most frequently reported approaches to coordinating care, and one study included GP views on out of hours’ care.

Identification, communication and education for professionals, families, patients, and the public was identified as the second key theme. Six studies focused on formal education including undergraduate, postgraduate, and continued professional development, some of which included the incorporation of new technology such as simulated learning for undergraduate nurses, and the online learning platform ECHO (Extension of Community Healthcare Outcomes) for community nurses. Studies assessing knowledge were undertaken with a range of stakeholders including the general public, nurses, speech and language therapists, and care home managers. Decision-making by staff, parents, and patients, and communicating with families were considered. One study focused on the education of staff to help Lesbian, Gay, and Bisexual (LGB) patients with palliative care needs and their families.

Other notable themes included methodology and evaluation, (14 studies), symptom management (12 studies), end of life care and bereavement (8 studies), and experiences, perceptions and needs (5 studies).

## Discussion

The findings from this study provide a comprehensive overview of Irish palliative care research undertaken over a five-year period (2012–2017), building upon a previous 10-year review of palliative care research in Ireland (2002–2012). The key findings demonstrate not only a continued upward trend in the quantity of palliative care research being undertaken in Ireland, but also developments in research impact as evidenced by the increase in publications in higher impact journals, increased collaborative working both nationally and internationally, some development in methodologies, and consolidation of key research themes such as research focusing on specific groups, services, and settings. Whilst palliative care research is on the increase in terms of quality and quantity, consideration of these findings in parallel with the recent reviews of palliative care in Scotland [10], South Asia [13], Sweden [11], and China [12] evidences that further progress is required.

### Building palliative care research capacity

Overall, 151 papers were identified in this 5-year period compared to 151 in the previous 10-year period, demonstrating an increase of 100%, and bringing research capacity in line with that of Scotland, which published 308 papers in a ten year period (2006–2015) [10]. In addition, whilst the majority of studies remained largely descriptive and needs based, there was an increase in review studies from 3% noted in previous review to 17%. This would suggest that researchers are increasingly seeing the value in collating and synthesising the evidence, to inform policy, practice, and future research studies. This increase in research activity can be attributed to various initiatives such as the establishment of the AIIHPC, PCRN [14]; growth in palliative care research groups; development of new Chairs in Palliative Care across the island (*n* = 5); increased collaboration with other specialist areas such as public health and gerontology; increase in the number of PhD research studentships and other research capacity building activities and workshops. This ongoing need for diverse and wide ranging research capacity building activities, from early career to senior investigators has been noted in the literature [17–19]. It is important that whilst the research to date may demonstrate an increase in the numbers of PhD studies, further consideration of developing a research career pathway and investment in post-doctoral opportunities is enhanced in order to ensure ongoing sustainability.

### Nature of research and perceived gaps

Whilst the research undertaken to date does address some of the identified palliative care research priorities in Ireland [20], for example, education and training for healthcare professionals (HCPs), providing care across multiple settings, and symptom management, there are also some clear gaps. These include areas such as the provision of out of hours’ services, ongoing support for carers, and advance care planning. Palliative care services and settings have been identified as a common research topic internationally [9, 10, 12, 13]. Whilst the inclusion of a mixture of settings within many studies reflects the reality of the patient experience, the focus on community settings is in line with international and national policy [7, 21]. In order to improve community based palliative care, some key areas for priority and consideration have been identified in the literature, including integration and timeliness of access to services, holistic management of pain and other symptoms, and compassionate and skilled providers [22]. In addition, future research needs to enhance the evidence base around different models of care, demonstrating not only effectiveness in terms of patient and family outcomes, but also cost effectiveness for practice, as evidence to date is inconclusive [23]. An emerging topic area identified from the review is the focus on cost effectiveness, which is a key strength and recognised as an important aspect of consideration for future intervention studies [24].

Another key aspect in relation to community based palliative care is the provision of out of hours’ care, which was also identified as a gap in Scottish palliative care research [10]. This review identified only one study which focused on out of hours’ service provision. This is noteworthy given that out of hours’ care was ranked as the most important priority area by both users, carers and health care professionals in a palliative care research priorities exercise [20]. Various challenges have been noted in practice, for example, GPs in England had very low confidence in their own ability to provide out of hours palliative care [25]. Therefore, this review demonstrates that whilst the palliative care research undertaken to date does focus on key services, populations and settings, there is both a research and clinical gap in relation to community based palliative care and specifically out of hours’ palliative care provision.

Symptom management was noted as a core theme, similar to previous reviews of palliative care research in Ireland, Scotland, Sweden, and China [9–12]. However, whilst symptom management is an important research topic, it was noted that research into physical symptoms dominated over psychological, social, or spiritual symptoms. This contrasts to the previous review in Ireland [9] which identified spiritual needs as a core theme. This identified lack of focus indicates the need for future research to address both psychological and physical aspects of symptom assessment and management in palliative care, within a multidisciplinary holistic focus.

Findings suggest there is an overreliance on reporting the HCP perspective and this may be indicative of significant challenges when undertaking research of this nature [26]. This contrasts to the person centred partnership approach currently being advocated within palliative care, as valuable insights into palliative care services are gained through triangulation of the patient, carer, and HCP perspective [27]. It is also in contrast to the findings of the Scottish review of palliative care [10], which found most studies recruited only patients. Therefore, it is vital that researchers continue to actively engage patients, carers, and families in developing and formulating research studies that will have an impact on practice and policy.

### Considerations of impact for palliative care research

Globally, palliative care research is often based on small, needs-based studies, according to data from Ireland, Scotland, Sweden, and South Asia [9–11, 13]. The current review demonstrated that, although research outputs increased, these were still mostly experiential and needs based studies. Gaps in interventional studies of effectiveness and cost effectiveness also existed. Only one RCT was identified in this review, highlighting a huge gap in empirical research. However, this may be explained due to the problematic issues surrounding RCTs within this vulnerable population. Using novel approaches to randomization, and incorporating multiple sources of evidence including qualitative research within the trial to capture processes and outcomes [28, 29] may increase the applicability of the RCT approach to palliative care. While the studies were still relatively small in size, over a third of studies collected data from Ireland, identifying an increase in collaborative working and networking across Ireland, By working collaboratively on a national and international level, research will translate into real world change at a practice and policy level for professionals, patients, and caregivers [30].

### Palliative care research funding

Funding is essential for research [1], and although palliative care in policy is seen as a priority, in reality palliative care research only receives a small portion of research funding. For example, less than 0.3% of cancer research funds are allocated to palliative care [31]. Within the UK and Ireland, the charitable sector has been a significant source of research funding [32, 33]. Whilst it is evident that the funding invested into the palliative care research networks in Ireland have led to an increase in the quantity of research, and an increase in national collaboration, additional funding for large scale, international collaborative projects would substantially advance the palliative care research field. At this time, however, questions exist around the potential implications of Brexit, including the availability of research funding sources and development of future research collaborations across Europe [34, 35].

### Strengths and limitations of the review

Limitations of this systematic review are acknowledged. One of the limitations was the difficulty in selecting key search terms to ensure all research in both ROI and NI were captured. Often, if research is conducted solely in NI, researchers may refer to “one region of the UK,” which would not show up when searching for Irish based studies. However, by including author details in the scope of the search, this should have captured anyone who affiliated themselves to a university or clinical site based in NI. Limiting the search to research where at least one author was from Ireland may have reduced the overall number of articles retrieved, however, this review was guided by the previous review conducted in Ireland and this approach was necessary to make the results comparable. This study was undertaken as a mapping review and whilst this afforded some key strengths in relation to other types of review such as scoping, the review did not include a quality appraisal of the studies. Given the diversity and range of the research studies review, quality appraisal would have been very complex and perhaps not very meaningful but it is acknowledged that further consideration of quality appraisal of the studies may have contributed to overall future assessment of the quality of the evidence for specific topic areas.

## Conclusions

This updated systematic mapping review identified a continued increase in palliative care research in Ireland with studies demonstrating increased collaborative working both nationally and internationally. Overall the quality and quantity of research increased from the previous review of Irish palliative care research (2002–2012). This can be attributed to significant investment in terms of research funding and collaborative networks. However, research gaps continue to exist, such as out of hours’ palliative care, psychological symptom control, limited use of experimental research design, and over reliance on HCP perspective. Key areas for attention include the need to continue to build research capacity across the continuum from doctoral to postdoctoral research opportunities, demonstration of the impact of the research on patient and family carer outcomes, service provision, and cost effectiveness. Whilst this follow up review has outlined progress specifically within the Irish palliative care context, there are clear lessons and opportunities for the global context. By undertaking such reviews at a country or regional level assists in developing a potential research database that can not only contribute to discussions at a national level but also inform the palliative care research community globally and by doing so, advance and improve provision of palliative care for patients and their families.

## Additional file

Additional file 1: Tabular summary of core themes and examples from the analysis. (DOCX 15 kb)

## Abbreviations

AIIHPC: All Ireland Institute of Hospice and Palliative care
EAPC: European Association for Palliative Care
ECHO: Extension of Community Healthcare Outcomes
GP: General Practitioner
HCP: Healthcare professionals
LGB: Lesbian, Gay, and Bisexual
NI: Northern Ireland
PCRN: Palliative Care Research Network
PCU: Palliative Care Unit
RCT: Randomised Control Trial
ROI: Republic of Ireland
UK: United Kingdom
WHO: World Health Organization
