# Supplementary material for: Examining palliative and end of life care research in Ireland within a global context: a systematic mapping review of the evidence
Source: BMC Palliat Care. 2018 Sep 27;17:109. doi: 10.1186/s12904-018-0364-7 (PMC6161399; doi:10.1186/s12904-018-0364-7)
Supplement: Supplementary file 1 — Tabular summary of core themes and examples from the analysis. (DOCX 15 kb) [file 12904_2018_364_MOESM1_ESM.docx]

Additional file 1: Summary of core themes and examples

| Core Themes | Sub-themes | Examples |
| --- | --- | --- |
| Specific Groups, Services, and Settings (n=70) | Cancer (n=12) | Experiences of patients (n=6), HCPs (n=2), carers (n=1), and a mix (n=3) across multiple settings using interviews or focus groups (n=6), questionnaires (n=2), a review (n=1), secondary data analysis (n=1) or a mix (n=2) |
|  | Dementia (n=12) | HCP resources, knowledge and attitudes of care home managers, attitudes of GPs regarding decision making, knowledge and experiences of pain management, barriers to care, attitudes of students |
|  | Parkinson’s Disease (n=6) | Knowledge and attitudes of HCPs, Patient and carer perceptions, MDT roles, exercise intervention pilot |
|  | Disability (n=6) | Develop a best practice model, inform and develop education for HCPs, explore needs and experiences of staff and patients, including children |
|  | Heart Failure (n=4) | Knowledge and attitudes of nurses, decision making, patient perceptions, use of advance care planning |
|  | Carers (n=5) | Psycho-educational intervention to mitigate distress, caregiver needs and experiences, benefits of family meetings |
|  | Children (n=3) | Resource development, needs of children with life limiting disabilities, pilot hospice at home for children |
|  | ALS (n=2) | Service engagement processes, patient journey |
|  | Renal Disease (n=1) | Patient views |
|  | Non-malignant (n=1) | Non-malignant referral patterns to hospital |
|  | Specific settings (n=11) | Hospices (n=5), Community/Home (n=1), Mixed (n=3), Review of organisational factors influencing transition between services (n=1) |
|  | Coordination of Services (n=6) | ACP (n=3), LCP (n=2), other (n=1) |
|  | Out of Hours services (n=1) | GP views relating to palliative patients and information transfer (n=1) |
| Identification, Communication and education (n=37) | Education, Training, and Knowledge (n=20) | HCPs (n=8), Students (n=4), General Public (n=2), Mixed (n=2), Not Applicable (Review) (n=1) |
|  | Communication (n=4) | Family experiences (n=2), HCPs and truth telling (n=1) |
|  | Decision making (n=3) | Nurses (n=1), parents (n=1), and patients (n=1) |
|  | Identification of needs (n=10) | General public (n=1), HCPs (n=3), expert academics and clinicians (n=4), and patients (n=1), identifying needs and priorities for research and clinical practice |
| Methodology and, Evaluation (n=14) | Methodology (n=2) | Producing a primary palliative care toolkit, intervention components for quality of life |
|  | Assessment (n=4) | Assessing staff perceptions of EOLC experiences, and symptom assessment in dementia care using a cancer assessment tool |
|  | Evaluation (n-3) | Pilot paediatric hospice at home service, a holistic assessment tool, and a dignity care intervention in the community |
|  | Economics (n=5) | Economic evidence on SPC consultation teams in the hospital, issues in identifying hospital costs of an intervention, economic cost of PC consultation on hospitals, costing palliative care services for children, review of costs of palliative care interventions |
| Symptom Management (n=12) | Delirium (n=6) | Literature reviews (n=5), developing an analytical framework (n=1), |
|  | Cachexia (n=1) | Consequences of honesty regarding terminal prognosis |
|  | Anorexia (n=1) | Evidence for use of corticosteroids |
|  | Frailty (n=1) | Review of theories |
|  | Edema (n=1) | Evaluation of palliative care edema service |
|  | Balance/Falls (n=1) | Determining incidence and risk factors |
|  | Mixed (n=1) | Reasons behind hospitalisation |
| End of Life Care and bereavement (n=8) | Last days of life (n=5) | Family member satisfaction, decision making for HCPs, review of studies using patient reported outcome measures of quality of EOLC, determinants of place of death, EoLC preferences |
|  | Bereavement (n=3) | Assessing bereavement care across Europe, parent experiences, effectiveness of creative arts activities, carer burden |
| Experiences, perceptions, and needs (n=5) | Mainly HCPs (n=2), and mixed (n=1) across hospital (n=1), hospice (n=1), or a mix of settings (n=1) | |
| Other (n=5) | Methicillin resistant Staphylococcus aureus (n=1), establishing a palliative care network (n=1), review of dignity conservation (n=1), PC policy analysis (n=1), PC in Ireland review (n=1) | |
